# Long-term Impact of Intravitreal Injections on the ocular surface; a 2-year follow-up study

**DOI:** 10.3389/fopht.2026.1829818

**Published:** 2026-07-17

**Authors:** Agni Malmin, Markus Vicente Tørud Olsen, Vilde Marie Thomseth, Inga-Britt Kjellevold Haugen, Tor Paaske Utheim, Vegard Asgeir Forsaa

**Affiliations:** 1 Department of Ophthalmology, Stavanger University Hospital, Stavanger, Norway; 2 Department of Clinical Medicine, University of Bergen, Bergen, Norway; 3 Department of Quality and Health Technology, University of Stavanger, Stavanger, Norway; 4 The Norwegian Association of the Blind and Partially Sighted, Oslo, Norway; 5 Department of Ophthalmology, Oslo University Hospital, Oslo, Norway

**Keywords:** anti-VEGF, dry eye disease, intravitreal injections, meibomian glands, ocular surface, povidone-iodine

## Abstract

**Background:**

Intravitreal injection (IVI) therapy is the most frequently performed intraocular procedure worldwide, with topical povidone-iodine (PVP-I) as the standard pre-injection antiseptic. However, PVP-I has been shown to exert cytotoxic effects on the ocular surface. The purpose of this study was to evaluate the impact of two years of serial anti-vascular endothelial growth factor (VEGF) IVI on ocular surface parameters.

**Methods:**

Patients with neovascular age-related macular degeneration (nAMD) receiving unilateral intravitreal anti-VEGF injections were examined at two time points, separated by a two-year interval. An aseptic protocol with PVP-I was applied prior to each injection. Tear meniscus height (TMH), bulbar redness (BR), and meibomian gland (MG) loss were assessed using the Oculus Keratograph 5M, with the fellow eye serving as control. For statistical analysis, the related-samples Wilcoxon signed-rank test was applied to non-normally distributed data, and the paired-sample Student’s t-test to normally distributed data.

**Results:**

Sixty patients (mean age, 78.6 ± 8.5 years; range, 55-96) were included. Between examinations, patients received a mean of 15.5 ± 6.5 IVI (range, 5-30). A significant increase in mean BR was observed in untreated fellow eyes compared with baseline measurements (1.68 ± 0.47 vs. 1.41 ± 0.46; *p* < 0.001). At follow-up, BR was significantly higher in fellow eyes than in treated eyes (*p* < 0.001), and this difference had increased over the study period. Median TMH increased significantly in untreated eyes (0.42 mm [IQR, 0.28–0.57] vs. 0.31 mm [IQR, 0.23–0.47]; *p* = 0.003), whereas the increase in treated eyes was not significant (*p* = 0.74). At follow-up, mean TMH did not differ significantly between treated and fellow eyes (*p* = 0.15). Both treated and untreated eyes showed significant MG loss after two years of serial IVI; however, no significant differences in mean MG loss were detected between eyes in either the upper or lower eyelid at follow-up.

**Conclusions:**

Eyes receiving repeated intravitreal anti-VEGF injections with preoperative PVP-I antisepsis were significantly less hyperemic than fellow untreated eyes, and this difference increased over two years of continued treatment. Potential mechanisms include a beneficial alteration of the ocular surface microbiome by PVP-I or an antiangiogenic effect of anti-VEGF therapy.

**Clinical Trial Registration:**

https://clinicaltrials.gov/study/NCT04458012, identifier NCT04458012.

## Introduction

Age-related macular degeneration (AMD) is a leading cause of visual impairment, affecting 196 million people globally (1, 2). The neovascular form, nAMD, is the primary cause of AMD-related vision loss (3) and is due to macular neovascularization mediated retinal edema, resulting in extensive damage to the photoreceptors. Treatment with intravitreal injections (IVI) of vascular endothelial growth factor (VEGF) inhibitors prevents vision loss and improves visual acuity, but due to the natural course of the disease the treatment must be repeated iteratively, usually every 4–6 weeks (4). To reduce the risk of endophthalmitis, topical povidone-iodine (iodine-polyvinylpyrrolidone, PVP-I) is routinely applied to the ocular surface before the injection (5). In a previous cross-sectional study, we showed that eyes receiving serial IVI exhibited less meibomian gland loss, higher and more physiological tear meniscus levels, and lower bulbar redness compared with fellow untreated eyes, suggesting a counterintuitive beneficial association between long-term IVI treatment and ocular surface status (6). We further found no evidence of chronic corneal epitheliopathy, and tear fluid analyses revealed reduced levels of proinflammatory cytokines in IVI-treated eyes (6, 7). These findings were unexpected given the well-documented cytotoxic effects of PVP-I on the ocular surface (8, 9). However, because our previous investigation assessed only a single time point, it could not determine whether these differences persist, diminish, or progress over time. The present study therefore serves as a two-year longitudinal follow-up, aimed at assessing how these ocular surface parameters evolve during continued IVI treatment. There are currently no prospective studies on the long-term effects of repeated IVI on the ocular surface, and given that IVI represent the most frequently performed intraocular procedure worldwide (10), improved understanding of potential surface effects is warranted.

## Materials and methods

Patients with nAMD in one eye treated with IVI of VEGF-inhibitors at the Department of Ophthalmology, Stavanger University Hospital, Norway, were included. For baseline measurements patients were enrolled from June 2020 throughout January 2022. Patients who were still receiving IVI at Stavanger University Hospital were re-examined between January 2022 and June 2023. The protocol has been described previously (6). The original inclusion criteria required a minimum of two previous IVI in one eye, ongoing treatment intervals ranging from 4 to 14 weeks, and a minimum interval of 4 weeks between the most recent IVI and the ocular surface assessment. The anti-VEGF agents administered included bevacizumab, aflibercept and ranibizumab. Exclusion criteria were eyelid disorders, trichiasis, entropion, ectropion, ptosis and eyelid movement disorder caused by facial paralysis. Moreover, patients using ocular medications at baseline were excluded, except for the use of artificial tear lubricants in both eyes. The IVI-protocol during the study period was as follows: Patients were pre-treated with 1 drop of tetracaine hydrochloride 1% ophthalmic solution (Minims, Bausch & Lomb, U.K. Inc.) followed by 1 drop of PVP-I 5% ophthalmic solution (Betadine; Alcon) to the ocular surface. The eye margin, lashes and periocular skin were cleaned with PVP-I in increasing concentric circles. Thereafter, an eye speculum was administrated, and a new drop of PVP-I was applied before the injection. After the IVI, there were no washout with saline irrigation or administration of topical antibiotics or anti-inflammatory drugs.

The study was approved by the Data Protection Officer at Stavanger University Hospital and the Regional Committee for Medical and Health Research Ethics (REK-ID 2019/832) and was performed in accordance with the tenets of the Declaration of Helsinki. The study was conducted based on oral and written consent from the patients and was registered at ClinicalTrials.gov (identifier, NCT04458012).

This study was registered retrospectively: the first participant was enrolled on 25.06.2020; the registration was submitted to ClinicaTrials.gov on 30.06.2020 and was publicly posted/approved on 02.07.2020, 1 week after first enrolment. The reason for registration after enrolment began was due to administrative delay. Although the study was registered after the inclusion of the first participants, the protocol, eligibility criteria, and outcome measures had been finalized prior to study initiation. The authors acknowledge the importance of prospective trial registration and recognize that registration prior to enrolment is best practice. One participant was examined before final registration of the study due to an administrative delay in the registration process. This participant fulfilled the same eligibility criteria as the remaining participants and was examined according to the same predefined protocol. No changes were made to the study design, outcome measures, or statistical analysis plan after this participant was included; therefore, the participant was retained in the analysis.

### Clinical measures

**Figure 1:**
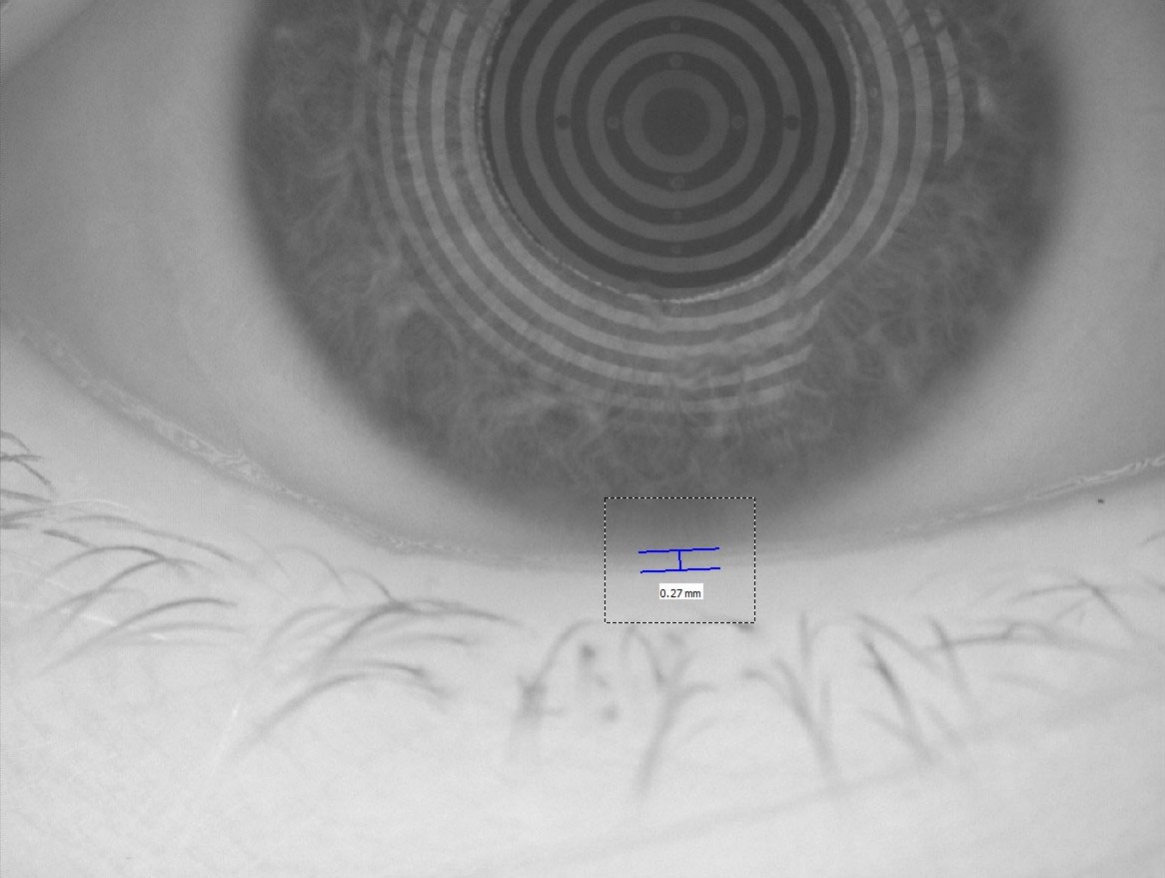
Tear meniscus height (TMH). Image captured with Oculus K5M. The TMH was manually measured as illustrated in the figure. With permission.

**Figure 2:**
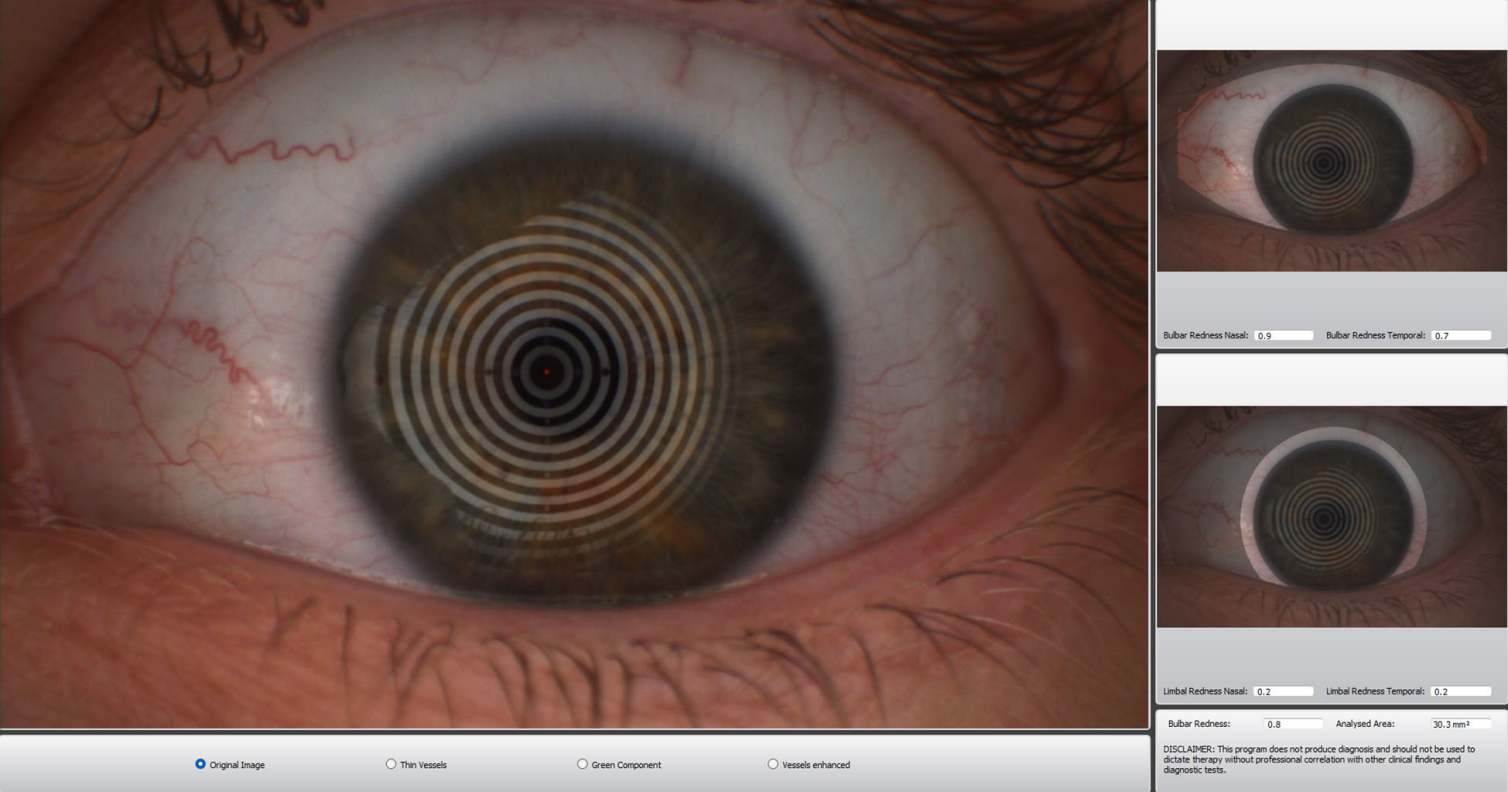
Measurement of bulbar redness using Oculus K5M. With permission.

**Figure 3:**
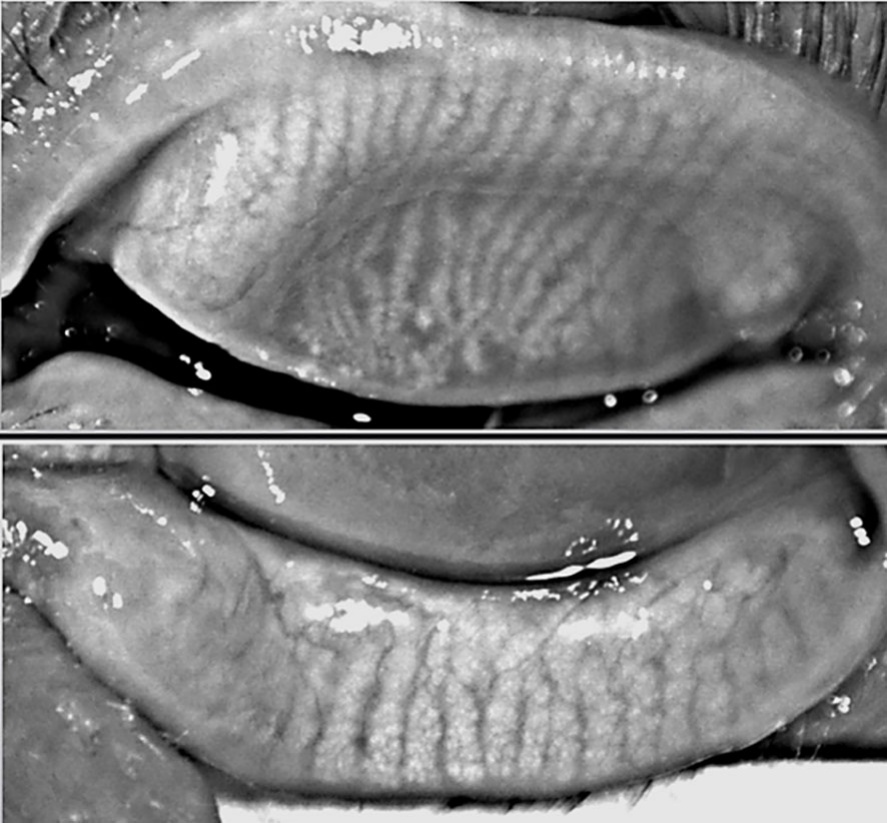
Infrared meibography of the meibomian glands in the upper and lower eyelid, performed with the Oculus K5M. With permission.

Ocular surface evaluation was performed using the Oculus Keratograph 5M (K5M) topographer (Oculus Optikgeräte GmbH, Wetzlar, Germany) in the following order: TMH, BR-score and meibography. TMH was measured inferior to the pupil center over the inferior eyelid margin (**Figure 1**). BR is an automated hyperemia grading system ranging from 0.0 to 4.0 (0 = minimal hyperemia, 4 = severe hyperemia) (**Figure 2**). The device captures standardized digital images of the bulbar conjunctiva, which are analyzed with proprietary algorithms to quantify redness based on relative red-channel activity. Consistent illumination ensures reliable imaging of the exposed bulbar conjunctiva. The software then calculates the BR-score by determining the percentage area occupied by conjunctival vessels relative to the total analyzed area. Meibography was performed in the upper and lower eyelid, and MG loss was manually determined using ImageJ software, calculated as (AreaDropout/AreaTarsal plate x 100%) (**Figure 3**). Meibography images were included in the analysis only when image quality was considered sufficient for reliable assessment of meibomian gland loss. Images were excluded if the meibomian glands could not be adequately visualized due to insufficient lid eversion, poor image quality, patient discomfort or pain during the procedure, or partial obscuration of the image by the examiner’s fingers. Upper eyelid meibography was more frequently excluded than lower eyelid meibography because adequate eversion of the upper eyelid was technically more difficult to obtain. The evaluators were masked to which eye was the receiving, and which was the control eye.

### Statistical analysis

Statistical analysis was performed using IBM SPSS Statistics 29.0.0.0 (SPSS Inc). The Shapiro-Wilk test was performed to examine the data for normal distribution. Normality was assessed on the distribution of paired differences. When the differences were normally distributed, a paired t-test was applied; otherwise, comparisons were made using the non-parametric Wilcoxon signed-rank test. Normally distributed data were demonstrated as the mean ± standard deviation, while non-normally distributed data were presented as the median with interquartile range (IQR). Ninety-five percent confidence intervals for the median differences are presented based on the Hodges-Lehmann estimator. P < 0.05 was considered statistically significant.

## Results

Sixty of the original ninety patients were reevaluated after two years. Exclusions and loss to follow-up were attributed to bilateral treatment (ten patients), treatment discontinuation (five patients), relocation (three patients), and death (ten patients). Two patients declined to participate.

Patients had a mean age of 78.6 ± 8.5 (range 55-96) years, received a mean number of 15.5 ± 6.5 (range, 5-30) IVI since baseline measurements and had received a total median of 36.5 IVI (range, 14-146; IQR 25-58) since treatment initiated (**Table 1**). The median time interval between the latest IVI was 4 weeks (range, 4-12) at baseline and 6 weeks (range, 4-20) at follow-up.

**Table 1:** Patient characteristics and number of intravitreal injections.

Patients	Total, n (%)	60 (100)
Injections	Female, n (%)	31 (52)
	Age, years ± SD (range)	78.6 ± 8.5 (55-96)
	Number (range)	36.5* (14-146; IQR 25-58)

Asterisk denotes median; SD, standard deviation; IQR, interquartile range.

**Figure 4:**
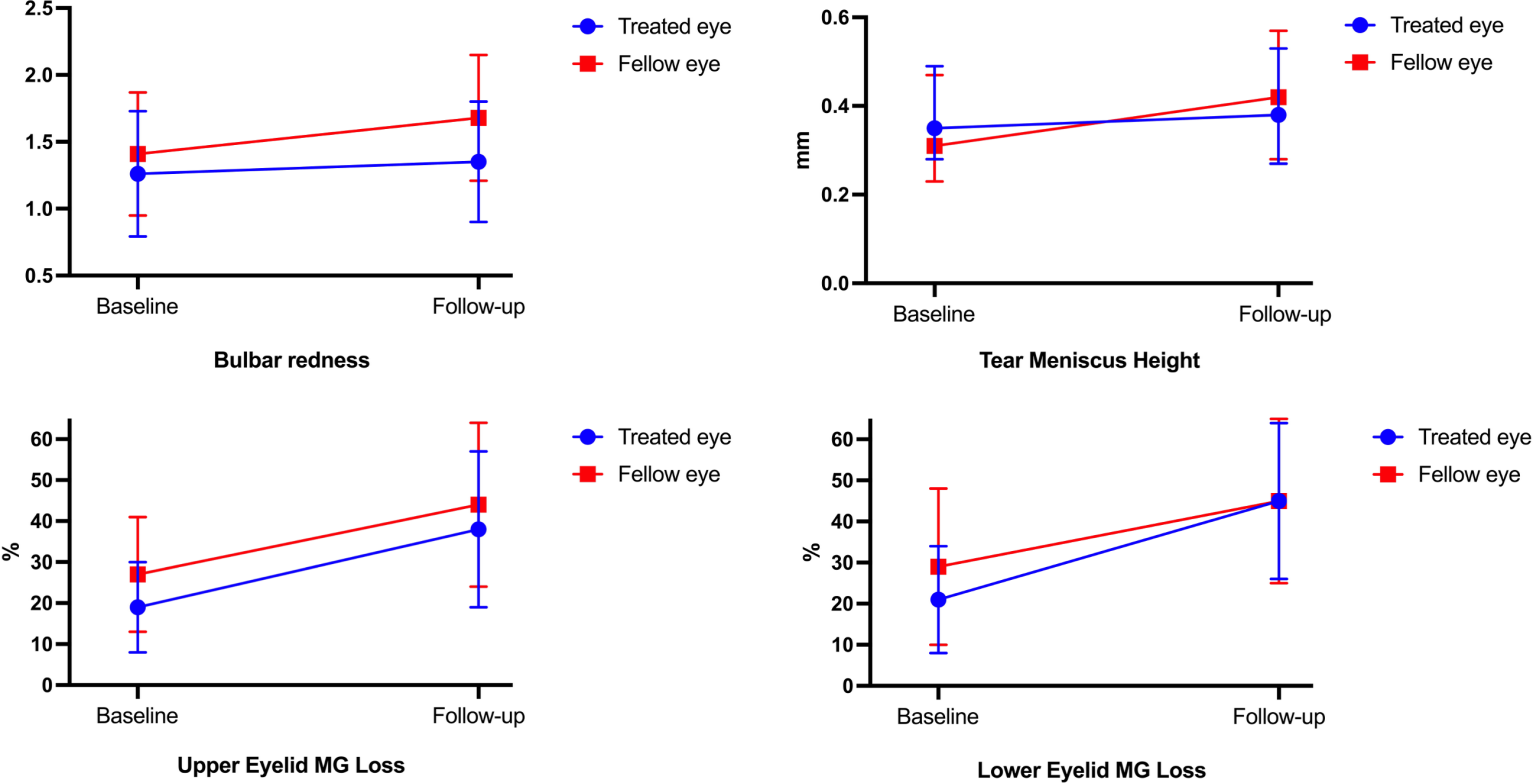
Baseline and 2-year follow-up values in treated and fellow eyes. Bulbar redness and meibomian gland loss are presented as mean values with standard deviations, whereas tear meniscus height is shown as median values with interquartile ranges. MG, meibomian glands.

We found a significant increase in mean BR in untreated fellow eyes compared to baseline measurements (baseline, 1.41 ± 0.46; follow-up, 1.68 ± 0.47, p < 0.001), but no difference in BR in treated eyes (baseline, 1.26 ± 0.47; follow-up, 1.35 ± 0.45, p = 0.22) after two years of IVI (**Table 2**; **Figure 4**). There was also a significant difference in BR between treated and fellow eyes at follow-up, with a mean BR of 1.35 ± 0.45 in the treated eye versus 1.70 ± 0.45 in fellow eyes, p<0.001 (**Table 3**).

**Table 2:** Measurements at baseline and 2-year follow-up for treated and fellow eyes.

Ocular surface parameter	Baseline	Follow-up	95% CI of the difference	n	p-value
Bulbar redness treated eye	1.26 ± 0.47	1.35 ± 0.45	0.09 (-0.05-0.23)	59	0.22
Bulbar redness fellow eye	1.41 ± 0.46	1.68 ± 0.47	0.27 (0.13-0.41)	59	<0.001
Tear meniscus height treated eye (mm)*	0.35 (0.28-0.49)	0.38 (0.27-0.53)	0.01 (-0.04-0.05)	60	0.74
Tear meniscus height fellow eye (mm)*	0.31 (0.23-0.47)	0.42 (0.28-0.57)	0.07 (0.03-0.11)	60	0.003
Upper eyelid MG loss treated eye (%)	19 ± 11	38 ± 19	19.0 (13.3-24.7)	23	<0.001
Upper eyelid MG loss fellow eye (%)	27 ± 14	44 ± 20	17.0 (9.5-24.5)	24	<0.001
Lower eyelid MG loss treated eye (%)	21 ± 13	45 ± 19	23.8 (18.5-29.2)	51	<0.001
Lower eyelid MG loss fellow eye (%)	29 ± 19	45 ± 20	16.8 (10.8-22.8)	54	<0.001

Mean values with standard deviations. Asterisks denote median values with interquartile ranges. CI, confidence interval; MG, meibomian gland.

**Table 3:** Ocular surface parameters in treated and fellow eyes at follow-up.

Ocular surface parameter	Treated eyes	Fellow eyes	95% CI of the difference	n	p-value
Bulbar redness	1.35 ± 0.45	1.70 ± 0.45	0.35 (0.21-0.48)	59	<0.001
Tear meniscus height (mm)	0.41 ± 0.18	0.44 ± 0.20	0.03 (-0.01-0.07)	60	0.15
Upper eyelid MG loss (%)	39 ± 17	43 ± 19	3.9 (-0.7-8.5)	54	0.095
Lower eyelid MG loss (%)	45 ± 20	45 ± 20	-0.6 (-5.7-4.5)	59	0.82

Mean values with standard deviations. CI, confidence interval; MG, meibomian gland.

There was a significant increase in median TMH in untreated eyes (baseline, 0.31 mm (IQR 0.23-0.47); follow-up, 0.42 mm (IQR 0.28-0.57), p=0.003) and a non-significant increase in TMH in treated eyes (baseline, 0.35 mm (IQR 0.28-0.49); follow-up, 0.38 mm (IQR 0.27-0.53, p=0.74) after two years of IVI (**Table 2**). At follow-up, mean TMH was 0.41 ± 0.18 mm in treated eyes and 0.44 ± 0.20 mm in the untreated fellow eye, not significantly different, p=0.15 (**Table 3**).

Regarding MG, there was a significant MG loss in the upper- and lower eyelid in both treated eyes (mean MG loss upper eyelid; baseline, 19 ± 11%; follow-up 38 ± 19%, p<0.001. Lower eyelid; baseline, 21 ± 13%; follow-up 45 ± 19%, p<0.001) as well as in untreated eyes (mean MG loss upper eyelid; baseline, 27 ± 14%; follow-up 44 ± 20%, p<0.001, lower eyelid; baseline, 29 ± 19%; follow-up 45 ± 20%, p<0.001) after two years of IVI. However, at follow-up, there was no difference in mean MG loss between the upper eyelid in treated eyes (39 ± 17%) and the fellow eyes (43 ± 19%, p=0.10), or between the lower eyelid in treated eyes (45 ± 20%) and the fellow eyes (45 ± 20%, p=0.82) (**Table 3**).

When calculating the differences in ocular surface parameters between treated and fellow eyes at baseline and again after two years of IVI, we found that the difference in BR had significantly increased, while the difference in THM and lower eyelid MG loss had significantly decreased (**Table 4**).

**Table 4:** Differences in ocular surface parameters between treated and follow eyes at baseline and follow-up.

Ocular surface parameter	Difference at baseline	Difference at follow-up	95 % CI difference	n	p-value
Bulbar redness	0.16±0.48	0.34±0.51	0.18 (0.00 5– 0.36)	58	0.04
Tear meniscus height (mm)*	-0.04 (-0.12-0.07)	0.03 (-0.09-0.09)	-0.05 (-0.1 - -0.01)	60	0.04
Upper eyelid MG loss (%)	6.4±9.6	4.2±20.0	-2.2 (-10.1 – 5.8)	22	0.58
Lower eyelid MG loss (%)	7.3±14.1	0.3±20.3	-7,0 (-13.5- -0,6)	51	0.04

Mean values with standard deviations. Asterisks denote median values with interquartile ranges. CI, confidence interval; MG, meibomian gland.

The values presented in **Tables 2**–**4** may differ slightly because the analyses are based on different patient subsets. **Table 2** includes only eyes with valid measurements at both baseline and follow-up for longitudinal within-eye comparisons. **Table 3** includes patients with valid measurements in both treated and fellow eyes at the follow-up examination for cross-sectional inter-eye comparisons. **Table 4** includes patients with valid paired inter-eye differences available at both baseline and follow-up.

## Discussion

In this two-year longitudinal follow-up study, we found that repeated IVI was associated with previously unreported long-term alterations of the ocular surface. We demonstrated that eyes treated with IVI were significantly paler than fellow untreated eyes, and the difference became more pronounced over the two-year treatment period due to progressive hyperemia in fellow untreated eyes. The findings are most likely explained by two mechanisms: inhibition of angiogenesis by anti-VEGF–therapy and the antimicrobial properties of PVP-I.

There was a significant increase in BR in untreated eyes two years after baseline, as opposed to treated eyes which did not increase in redness. Ageing is associated with chronic inflammation due to a low-grade, persistent activation of the immune system. Ageing is also associated with decreased tear film stability, decreased tear production and an increase in proinflammatory cytokines. Additionally, age is a significant risk factor for alteration of the microbiome on the ocular surface (21). The persistent low bulbar redness in treated eyes could indicate a protective effect of repeated anti-VEGF or PVP-I against age-related ocular surface changes.

In AMD, neovascularization is driven by the recruitment of immune cells and the release of pro-inflammatory and pro-angiogenic cytokines—such as VEGF—that stimulate abnormal blood vessel growth (11). Anti-VEGF is also used in the treatment of corneal neovascularization, and studies have found an effect of anti-VEGF both when applied subconjunctivally and topically (12). When anti-VEGF is administered intravitreally, there might occur a reflux of medication through the injection canal when extracting the needle after the procedure, either to the subconjunctival space or to the ocular surface. This would lead to inhibition of VEGF across the ocular surface, which in turn would cause a reduction in conjunctival vascularization when given repeatedly. Additionally, anti-VEGF may also reach the ocular surface by diffusion from the vitreal space through the sclera, but the extent of this movement is generally low (13, 14). It would be of interest to quantify anti-VEGF concentrations on the ocular surface immediately post-IVI, for example through serial tear sampling to monitor changes over time.

The ocular microbiota plays a key role in maintaining the ocular surface microenvironment in health and disease (15). As previously described, we routinely used povidone iodine (PVP-I) 5% prior to the procedure to reduce the risk of endophthalmitis. We hypothesize that this alters the microbial environment and could be the cause of the difference in redness between the two eyes. PVP-I is a complex of iodine and a solubilizing carrier. A small amount of free, bactericidal iodine is constantly released from the complex and penetrates the bacterial cell, resulting in rapid cell death (16). PVP-I is effective in eliminating bacteria like *Cutibacterium acnes*, coagulase-negative staphylococci and *Corynebacterium* species, which are found in greater numbers in dry eye disease (17, 18), the latter found especially in older individuals (19). In a randomized placebo-controlled study exploring the effect of PVP-I for nasal decolonization PVP-I reduced the bioburden and eliminated pathogens without disruption of the microbiome (20). Thus, we hypothesize that PVP-I causes a reduction in bulbar redness by removing pathogenic microbes on the ocular surface, reducing bacterial load and dysbiosis, contributing to a reduction of inflammation and thereby a paler conjunctiva. Although we did not measure the microbiome, this could be worth exploring in future studies.

We found a significant MG loss in upper- and lower eyelids in both treated and untreated eyes at two years compared to baseline measurements. Although MG loss appears to have accelerated in eyes receiving IVI, there were no significant differences in MG loss between treated eyes and fellow eyes at follow-up. Several studies have examined the effect of ageing on meibomian glands. With increasing age, structural changes including decreased diameter and density, and an increase in meibomian gland dropout and obstruction occur (22). Regarding TMH, we found a significant increase in TMH in untreated eyes at follow-up and no difference in treated eyes after two years of IVI. However, there was no difference in TMH at follow-up between treated and untreated eyes. Previous studies have found a decrease in tear osmolarity after serial IVI-treatment (23), likely due to a stimulation of the lacrimal-functional unit in the treated eye. There are well-established mechanisms which activate lacrimal stimulatory nerves on both the ipsilateral and contralateral side, for instance the trigeminal autonomic reflex (24). Thus, we hypothesize that reflex mechanisms, like the trigeminal autonomic reflex, could be an explanation for our findings. Increased eyelid laxity with age can also contribute to this finding.

Strengths of the study are the prospective design, as well as the patients being their own controls, which minimizes environmental and medical related biases. Moreover, we did not apply topical antibiotics or anti-inflammatory drugs post-injection, which would have introduced a potential bias of ocular surface inflammation. Bulbar redness is a hyperemia measurement automatically calculated by the device, without any subjective evaluation. The Oculus K5M topographer is an objective and reliable method for scoring BR, and is shown to have the highest reproducibility when comparing scoring modalities for hyperemia (25).

A limitation of the study is that patients were not asked to report their use of topical eye medication at follow-up; therefore, information on unilateral topical therapy or possible blepharitis treatment during the follow-up period was unavailable. Although artificial tear lubricants were permitted only when used bilaterally, the timing of the last instillation before ocular surface examination was not systematically recorded, and no standardized washout period was applied. Recent use of tear substitutes may influence tear meniscus height and bulbar redness and may therefore have introduced variability in the measurements. However, as only bilateral use was permitted, this is unlikely to fully account for inter-eye differences between treated and fellow eyes. Other limitations to the study are lack of multi-observer evaluation of MG loss, which was evaluated by one masked grader through semi-automatic measurements. The term baseline refers to the initial set of measurements collected at the start of a study, prior to intervention or follow-up, and it serves as the reference point for subsequent comparison. Although this was a prospective study, the participants were not treatment-naïve, as the study eye already had received multiple IVI at baseline. Moreover, the sample size varied between outcome measures because some meibography images, particularly of the upper eyelids, were of insufficient quality for reliable assessment. This reduced the number of included observations and may have decreased statistical power for meibomian gland analyses. Therefore, results related to meibomian gland loss, especially in the upper eyelid, should be interpreted with caution.

Tetracaine hydrochloride is a local anesthetic that acts by blocking sodium channels (26). *In vitro* studies have demonstrated dose- and time-dependent cytotoxic effects on corneal epithelial cells (27) and reduced tear film break-up time has been reported in horses (28). In contrast, a single 0.5% dose did not induce detectable alterations in corneal microvilli or epithelial structure in a rabbit model evaluated by scanning electron microscopy (29). Kang et al. further showed that tetracaine exhibits antimicrobial activity only after prolonged exposure, with no effect observed following exposure under two minutes (30). The ocular surface consequences of repeated tetracaine exposure, particularly regarding epithelial integrity and the ocular microbiome, remain uncertain. Therefore, tetracaine cannot be fully excluded as a contributing factor to our findings.

Another limitation is that the present follow-up focused on ocular surface parameters that showed relevant differences in our previous cross-sectional study. This approach may introduce selection bias toward outcomes already known to be responsive and limits the ability to draw conclusions about ocular surface parameters not included in the current analysis. Therefore, the findings should be interpreted as longitudinal follow-up of selected previously identified parameters rather than as a comprehensive assessment of all possible long-term ocular surface effects of repeated IVI.

The clinical relevance of the observed differences should be interpreted with caution. Although statistically significant changes were detected, particularly in bulbar redness, the study did not include patient-reported symptom scores. Therefore, it remains uncertain whether the measured differences are of sufficient magnitude to affect ocular comfort, visual quality, or clinical management. The findings should consequently be interpreted primarily as objective, device-detected ocular surface changes rather than as evidence of clinically meaningful benefit. The effects of anti-VEGF therapy and pre-injection PVP-I antisepsis cannot be separated, as treated eyes were exposed to both components whereas fellow eyes were exposed to neither. Thus, observed differences reflect associations with repeated IVI in routine practice rather than effects of either component alone. Although both exposures represent biologically plausible explanations for the observed reduction in bulbar redness, their relative contributions cannot be determined from this study design.

The impact of IVI on the ocular surface remains controversial, with differing results between studies (31–36). Eye departments have divergent protocols and traditions for IVI regarding cleaning, disinfection and postoperative antibiotic or anti-inflammatory prophylaxis, which may explain the various findings. Moreover, the ocular surface is a complex composition and significant differences in the microbiota have been found in patients from different geographical locations (19).

## Conclusion

Eyes receiving repeated intravitreal injections were significantly paler than fellow untreated eyes, and this difference increased after two years of continued IVI treatment. This indicates a reduction of inflammation or an inhibition of vascularization in the conjunctiva. Possible explanations of the difference in redness are alteration of the ocular surface microbiota in a beneficial manner due to pre-injection povidone-iodine or an antiangiogenic effect from repeated VEGF-inhibitors. A contralateral increase in tear meniscus height could be explained through the trigeminal reflex, while bilateral meibomian gland loss might be due to ageing.

## Data Availability

The original contributions presented in the study are included in the article/supplementary material. Further inquiries can be directed to the corresponding author.
